# Association between protein intake, serum albumin and blood eosinophil in US asthmatic adults

**DOI:** 10.3389/fimmu.2024.1383122

**Published:** 2024-05-21

**Authors:** Jun Wen, Jing Xia, Qingliu He, Mohan Giri, Shuliang Guo

**Affiliations:** ^1^ Department of Respiratory and Critical Care Medicine, The First Affiliated Hospital of Chongqing Medical University, Chongqing Medical University, Chongqing, China; ^2^ Department of Respiratory and Critical Care Medicine, The Third Affiliated Hospital of Chongqing Medical University, Chongqing Medical University, Chongqing, China; ^3^ Department of Urology, The Second Affiliated Hospital of Fujian Medical University, Fujian Medical University, Fujian, China

**Keywords:** protein intake, albumin, eosinophil, asthma, National Health and Nutrition Examination Survey (NHANES), XGBoost

## Abstract

**Background:**

Presently, numerous studies have indicated that protein consumption and levels of blood albumin serve as important biomarkers for a range of respiratory illnesses. However, there have been few investigations into the correlation between protein consumption, serum albumin, and asthma.

**Methods:**

Our analysis incorporated 2509 asthmatics from the 2011–2018 NHANES dataset. The investigation employed three linear regression models and XGBoost model to investigate the potential link between protein intake, serum albumin levels, and blood eosinophil counts (BEOC) in patients with asthma. The trend test, generalized additive model (GAM), and threshold effect model were utilized to validate this correlation. As well, we undertook stratified analyses to look at the correlation of serum albumin with BEOC among distinct populations.

**Results:**

In the univariable regression model, which did not account for any covariates, we observed a positive correlation between protein intake and BEOC. However, univariable and multivariable regression analyses all suggested a negative connection of serum albumin with BEOC in asthma populations. In Model C, which took into account all possible factors, BEOC dropped by 2.82 cells/uL for every unit increase in serum albumin (g/L). Additionally, the GAM and threshold effect model validated that serum albumin and BEOC showed an inverted U-shaped correlation.

**Conclusion:**

Our investigation discovered there was no independent link between asthmatics’ protein intake and BEOC. However, we observed an inverted U-shaped relationship between serum albumin levels and BEOC, suggesting a possible relationship between the overall nutritional status of asthmatics and immune system changes. Our findings provide new directions for future research in the field of asthma management and therapy.

## 1 Introduction

Asthma, which manifests as a clinical syndrome of inflammation, bronchial hyperresponsiveness, and reversible airflow obstruction, is a prevalent chronic airway disease (1). The prevalence of asthma has witnessed a significant rise in several nations over the last few decades. At present, the prevalence estimates for asthma among children and adults in the United States stand at 10.9% and 18.5%, respectively (2). Asthma was responsible for around 1.6 million visits to emergency departments and 183,000 hospitalizations in the United States in 2017, according to the survey (3). This caused a significant economic burden and a considerable number of missed school days.

Airway eosinophilia is a frequent clinical manifestation of asthma, a chronic inflammatory disease of the airways (4). Eosinophils play a role in multiple pathological processes, including airflow obstruction in asthma and chronic airway remodeling (5, 6). These processes include smooth muscle hypertrophy, neural plasticity, epithelial injury, and impaired tissue repair. The blood eosinophil count is a widely recognized and readily available biomarker that is of paramount importance in the management of asthma (7, 8). The correlation between elevated blood eosinophil levels and compromised disease control, as well as an increased susceptibility to severe asthma exacerbations, has been established by a multitude of studies (9–13). Additionally, blood eosinophils can be utilized to predict the response of asthma therapy and guide treatment decisions (14–17). In conclusion, blood eosinophils are an essential biomarker for asthma management and play a critical role in the onset, progression, and treatment of asthma.

Albumin is the predominant protein found in serum, making up over 60% of all blood proteins (18). It is solely produced in the liver and then released into the bloodstream. Serum albumin, with a half-life of 19 days, is crucial for regulating several physiological processes. Keeping the acid-base balance, transporting important molecules (like long-chain fatty acids, hormones, bilirubin, metal ions, etc.) through the bloodstream and to organs, stopping platelet function, Keeping the acid-base balance, transporting important molecules (like long-chain fatty acids, hormones, bilirubin, metal ions, etc.) through the bloodstream and to organs, stopping platelet function, keeping vascular permeability, and managing colloid osmotic pressure are some of these jobs (19). Furthermore, it demonstrates antioxidant properties and the ability to trap free radicals (20). Furthermore, serum albumin serves as an established clinical indicator for malnutrition (21). Asthmatic persons frequently experience malnutrition, which adversely affects their quality of life, exacerbation risk, duration of hospitalization, and overall healthcare costs (22, 23).

Recent research has shown that there is a correlation between low levels of albumin in the blood (hypoalbuminemia) and an extended duration of hospitalization in patients with acute exacerbations of chronic obstructive pulmonary disease (COPD) (24, 25). In individuals suffering from acute pulmonary embolism, low levels of serum albumin continue to serve as indicators of long-term mortality (20). However, there have been few investigations conducted to explore the connection between protein status and asthma thus far. Consequently, we utilized data from the National Health and Nutrition Examination Survey (NHANES) 2011–2018 cycles to examine the connection between protein intake, serum albumin and BEOC in patients with asthma.

## 2 Materials and methods

### 2.1 Study data and population

The data for this investigation were collected from the NHANES public database, which was a part of the Centers for Disease Control and Prevention (CDC) in the United States. The NHANES database was responsible for collecting vital and health statistics for the country. The NHANES survey was conducted using a sophisticated multistage stratified sample design to ensure that a diverse and accurate representation of the US population was obtained. The program included interviews, medical exams, and laboratory tests. The collected data was used to analyze the correlation between nutritional status and promoting health and preventing diseases. All participants underwent the necessary procedures for obtaining informed consent and comprehensive health tests. The NHANES study protocol received approval from the Research Ethics Review Board of the National Center for Health Statistics.

**Figure 1:**
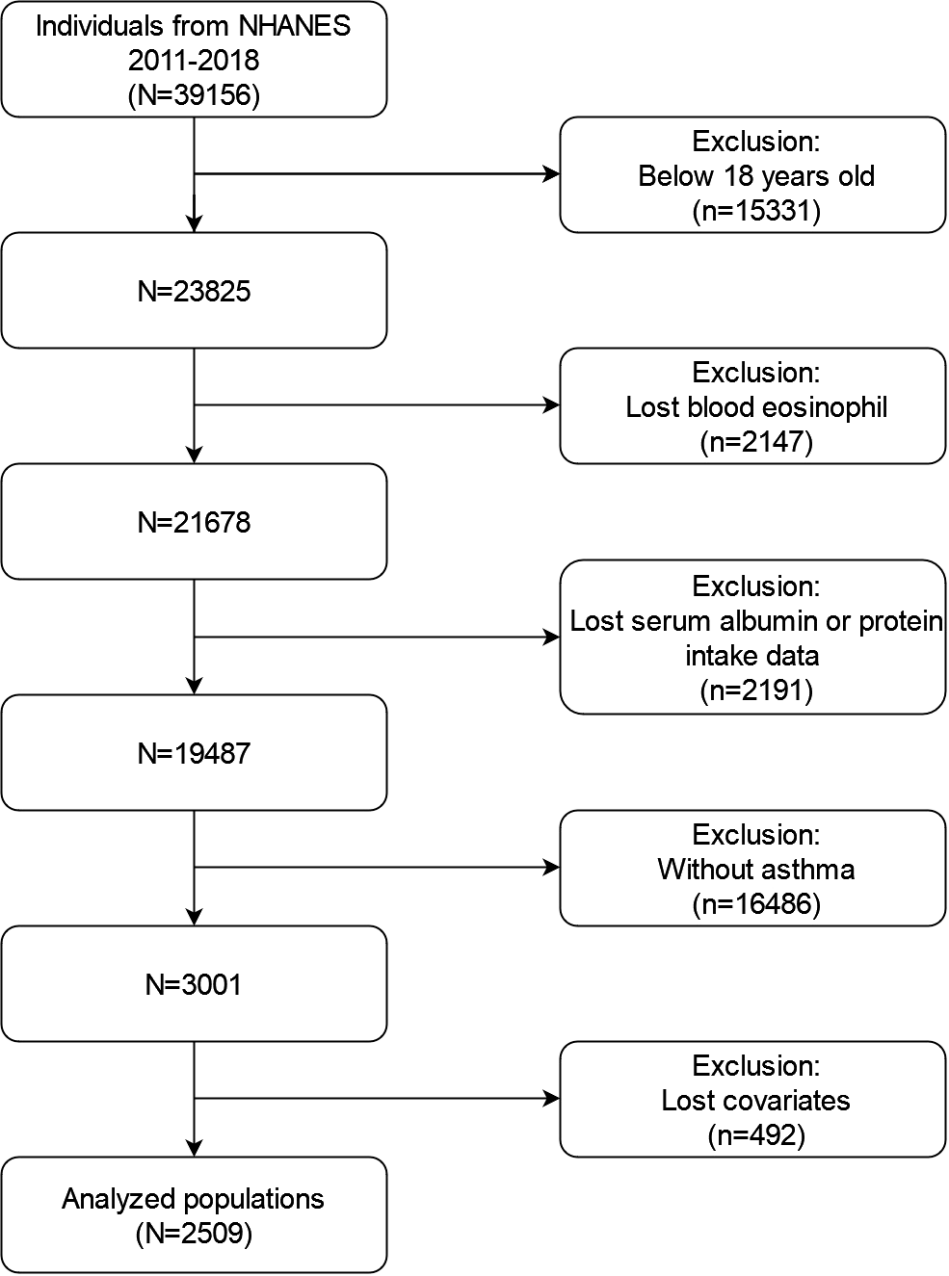
Flowchart for the studied individual’s selection process.

Between 2011 and 2018, the NHANES amassed a total of 39156 individuals of data. The following individuals were excluded from our investigation: (1) under 18 years old (n=15331); (2) missing BEOC (n=2147); (3) missing serum albumin or protein intake data (n=2191); (4) non-asthmatics (n=16486); (5) missing over one of following covariates (n=492): education, marriage, poverty to income ratios (PIR), body mass index (BMI), smoking state, alcohol intake, hypertension, diabetes, liver condition, COPD, cancer history, aspartate aminotransferase (AST), alanine aminotransferase (ALT), serum creatinine, serum globulin, serum total protein, and urine albumin, steroid use. At last, a large, nationally representative group (n=2509) of asthmatic adults in the United States was collected for our investigation. The screening procedure’s flowchart appears in **Figure 1**.

### 2.2 Measurement of serum albumin and blood eosinophil counts

The bichromatic digital endpoint technique (DcX800) is applied in order to determine the concentration of albumin in the serum. Throughout the procedure, bromcresol purple (BCP) reagent and albumin combine to form a complex. At 600 nanometers, the system logs any fluctuations in absorbance. The change in absorbance is directly proportional to the quantity of albumin present in the sample. The dietary protein intake of the participants was assessed using a 24-hour dietary recall approach. A skilled technician requested information regarding the specific kinds and amounts of food and medication consumed in a single day. This information was then recorded in the NHANES computer-assisted dietary survey system. A Beckman-Coulter MAXM analyzer was utilized to perform a complete blood count and 5-part differential on whole blood samples. For use in *post hoc* analyses, cell counts of lymphocytes, monocytes, segmented neutrophils, eosinophils, and basophils (1000 cells/uL) were obtained using the 5-part differential measure.

### 2.3 Covariates and asthma assessment

We incorporated numerous covariates in our investigation to lessen the potential impact of confounding variables. These factors were looked at: sex, age, race, education, PIR (low, middle, and high), marriage, BMI, history of smoking, alcohol use, high blood pressure, diabetes, liver disease, COPD, cancer, recent use of steroid drugs, AST, ALT, serum creatinine, serum globulin, serum total protein, and urine albumin. When people came in for their visit, standard surveys were used to confirm whether they had asthma. The intended evaluation question was: “Have you ever received a diagnosis of asthma from a doctor?” People who said “yes” were identified as having asthma.

### 2.4 Statistical analysis

The statistical analyses were accomplished via the R program (version 4.2.0). A p-value below 0.05 implied statistical significance. To address the complex sampling design of the NHANES, sample weights were incorporated. The BEOC was initially converted into quartiles. The p-value for categorical variables was calculated via the chi-square test, while for continuous variables, the Kruskal-Wallis rank sum test was deployed. Three distinctive linear regression models (Model A, Model B, and Model C) were utilized to evaluate the connection between protein intake, serum albumin, and BEOC while accounting for different covariates. And we employed trend analysis, the generalized additive model (GAM), and the threshold effect model to test the linearity or nonlinearity of the connection of serum albumin with BEOC. If a non-linear correlation was detected, a two-piecewise linear regression model was utilized to ascertain the threshold impact of serum albumin levels on BEOC. When the relationship between serum albumin and BEOC was obvious in a smoothed curve, the recursive technique automatically predicted the inflection point at which the greatest model likelihood would be adopted. As well, stratified analyses were conducted to investigate the connection of serum albumin with BEOC in various groups. Lastly, our investigation applied the XGBoost algorithm model to evaluate the relative significance of different indicators in connection to the impact of BEOC.

## 3 Results

### 3.1 Baseline characteristics of study individuals based on BEOC quartiles

We utilized BEOC quartiles to subdivide the weighted characteristics of the 2509 adults with asthma who participated in our investigation (1031 men and 1478 women) (**Table 1**). The research sample comprised non-Hispanic white individuals, with an average age of 47.1 years, representing the designated participants’ demographic. The distributions of sex, age, race, BMI, hypertension, ALT, serum creatinine, serum total protein, and serum albumin varied significantly between the quartiles of BEOC. However, the distributions of education, marriage, PIR, smoking, alcohol consumption, protein consumption, diabetes, liver condition, COPD, cancer, steroid drug use, AST, serum globulin, and urine albumin did not differ significantly between the quartiles of BEOC. Participants whose BEOC was in the lowest quartile had elevated levels of serum total protein and serum albumin, in addition to lower values of age, BMI, ALT, and serum creatinine (p < 0.05), in comparison to the other groups.

**Table 1:** The weighted characteristics of the study population were analyzed based on quartiles of BEOC.

	Q1 (0)	Q2 (100)	Q3 (200)	Q4 (300-2200)	P value
Sex (%)					0.0040
Male	33.96	34.15	40.38	45.51	
Female	66.04	65.85	59.62	54.49	
Age (years)	45.93 ± 1.82	43.39 ± 0.74	46.14 ± 0.83	46.97 ± 0.71	0.0021
Race (%)					0.0277
Mexican American	4.96	4.55	5.85	6.87	
Other Hispanic	5.58	5.75	6.07	6.41	
Non-Hispanic White	63.35	69.54	68.9	68	
Non-Hispanic Black	23.44	12.42	10.5	10.97	
Other Race	2.67	7.74	8.68	7.75	
Education (%)					0.2390
Below high school	14.65	10.31	13.29	12.61	
High school	17.18	19.16	20.39	23.62	
Above high school	68.17	70.52	66.32	63.78	
Marriage (%)					0.3450
Married	41.57	52.52	48.72	52.76	
Single	54	39.74	43	39.73	
Living with a partner	4.43	7.74	8.28	7.51	
PIR	2.51 ± 0.23	2.91 ± 0.09	2.74 ± 0.11	2.79 ± 0.1	0.1540
BMI (kg/m2)	29.32 ± 1.01	29.54 ± 0.32	30.85 ± 0.49	31.54 ± 0.42	0.0005
Smoking (%)					0.0779
Smoker	51.1	43.63	45.11	52.23	
Non-smoker	48.9	56.37	54.89	47.77	
Alcohol intake (gm)	14.62 ± 3.62	12.13 ± 1.41	12.31 ± 1.98	12.75 ± 1.69	0.8919
Protein intake (gm)	83.09 ± 9.7	78.76 ± 1.75	83.92 ± 1.98	84.72 ± 2.2	0.0780
Hypertension (%)					0.0031
Yes	38.55	30.04	37.49	41.05	
No	61.45	69.96	62.51	58.95	
Diabetes (%)					0.1215
Yes	15.91	8.12	12.93	13.89	
No	80.94	88.89	84.79	83.43	
Borderline	3.15	2.99	2.29	2.68	
Liver condition (%)					0.2336
Yes	4.29	4.45	7.09	4.6	
No	95.71	95.55	92.91	95.4	
COPD history (%)					0.1199
Yes	15.97	7.63	11.24	9.51	
No	84.03	92.37	88.76	90.49	
Cancer history (%)					0.4814
Yes	13.36	10.12	11.03	13.42	
No	86.64	89.88	88.97	86.58	
Steroid drugs use (%)					0.1958
Yes	20.74	14.22	15.91	18.77	
No	79.26	85.78	84.09	81.23	
AST (U/L)	22.95 ± 1.03	24.86 ± 0.89	25.59 ± 0.61	24.33 ± 0.54	0.1223
ALT (U/L)	24.27 ± 1.75	35.87 ± 1.25	35.45 ± 1.55	34.36 ± 1.16	<0.0001
Serum creatinine (umol/L)	73.4 ± 2.15	72.8 ± 0.73	77.57 ± 0.88	79.23 ± 1.1	<0.0001
Serum globulin (g/L)	28.58 ± 0.63	28.03 ± 0.19	28.06 ± 0.26	28.53 ± 0.21	0.0827
Serum total protein (g/L)	71.39 ± 0.57	70.63 ± 0.19	70.09 ± 0.23	70.44 ± 0.21	0.0367
Urine albumin (mg/L)	29.82 ± 8.75	31.99 ± 8.21	31.67 ± 11.05	33.97 ± 6.18	0.9830
Serum albumin (g/L)	42.81 ± 0.64	42.6 ± 0.15	42.03 ± 0.19	41.9 ± 0.15	0.0009

Continuous and categorical variable were displayed individually as weighted means ± SD or proportions. Q1-Q4: BEOC had been classified into quartiles. gm, gram; mg, milligram; mcg, microgram.

### 3.2 Association between protein intake, serum albumin and BEOC

We applied both univariable and multivariable linear regression models to investigate the connection among asthma patients’ protein intake, serum albumin, and BEOC. Only Model A, which did not account for any covariates (**Supplementary Table 1**), revealed a positive correlation of protein intake with BEOC. Nevertheless, we all identified with statistical significance in Models A, B, and C the inverse correlation of serum albumin with BEOC (**Table 2**). In Model C, which controlled for all covariates, BEOC decreased by 2.82 cells/uL for each extra unit of serum albumin (g/L). Furthermore, the outcomes of the trend test revealed statistical significance in Models A and B (p for trend < 0.05). However, no statistical significance was observed in the trend test of Model C (p for trend> 0.05), which suggesting a potential non-linear correlation of serum albumin with BEOC.

**Table 2:** Association between serum albumin and BEOC in asthmatics.

	Model A	Model B	Model C
	β (95% CI) P value	β (95% CI) P value	β (95% CI) P value
Serum albumin (g/L)	-2.87 (-5.14, -0.60) 0.0161	-4.64 (-7.03, -2.24) 0.0004	-2.82 (-5.52, -0.13) 0.0486
Serum albumin quartile			
Q1 (21-39)	Reference	Reference	Reference
Q2 (40-41)	1.20 (-30.03, 32.43) 0.9404	-5.03 (-35.75, 25.68) 0.7493	0.94 (-32.41, 34.29) 0.9563
Q3 (42-43)	-17.27 (-43.98, 9.43) 0.2098	-28.39 (-54.99, -1.79) 0.0414	-15.09 (-52.60, 22.41) 0.4368
Q4 (44-52)	-26.98 (-49.20, -4.75) 0.0206	-43.04 (-66.30, -19.79) 0.0007	-22.21 (-74.42, 30.00) 0.4115
P for trend	0.0032	0.0001	0.2895

Model A controlled for none. Model B controlled for sex, age and race. Model C controlled for sex, age, race, education, marriage, PIR, BMI, smoking, alcohol intake, protein intake, hypertension, diabetes, liver condition, COPD, cancer history, steroid drugs use, AST, ALT, serum creatinine, serum globulin, serum total protein and urine albumin. Q1-Q4: Seum albumin was grouped by quartile.

### 3.3 Generalized additive model and threshold effect model

**Figure 2:**
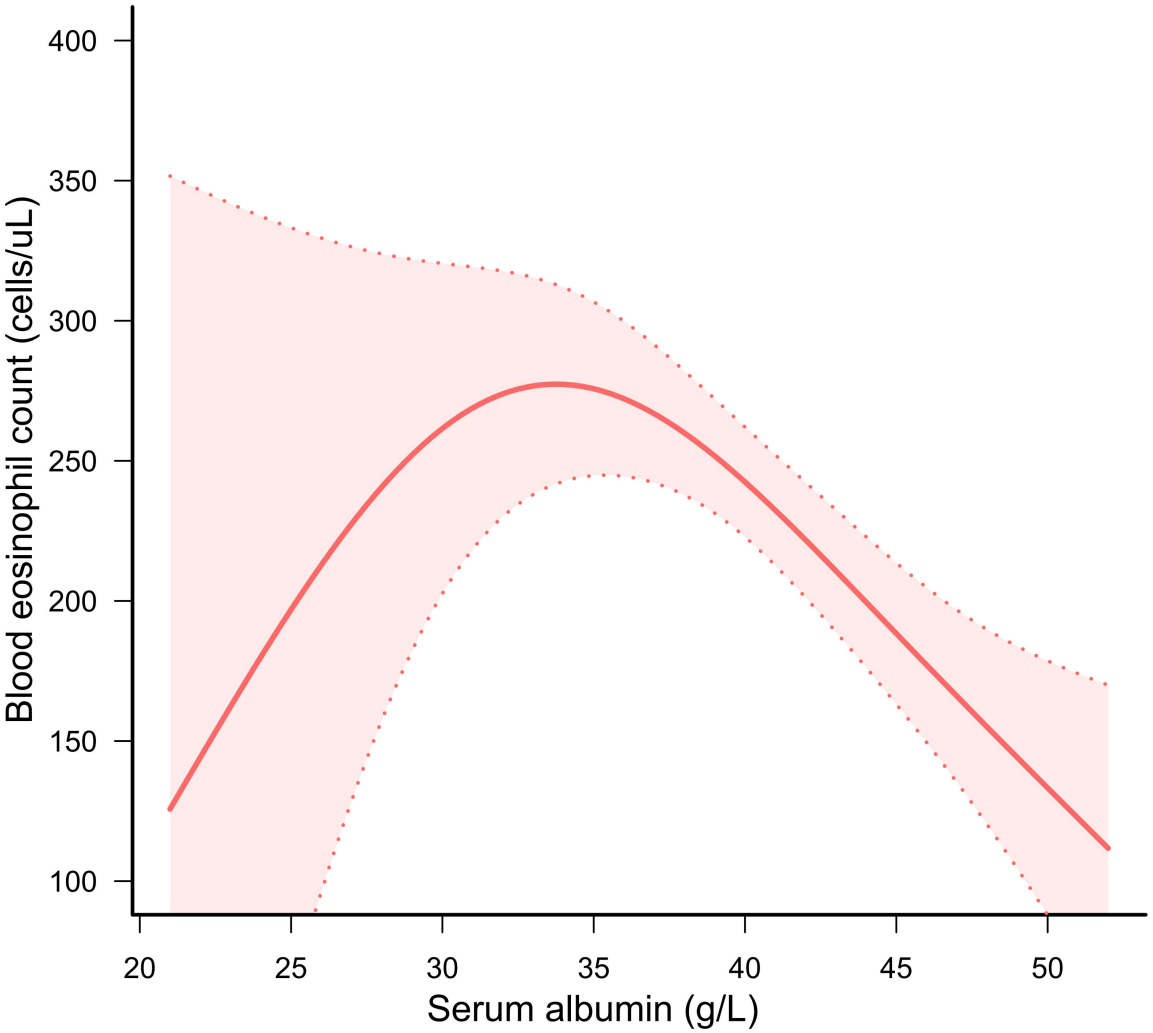
A solid red line displayed the correlation of serum albumin with BEOC. A red area indicated suitable 95% confidence ranges.

The GAM and threshold effect models were highly effective in identifying whether the correlation demonstrated linearity or nonlinearity. We employed GAM to generate a smooth and continuous curve based on Model C. This enabled us to ascertain if there was a nonlinear correlation between serum albumin and BEOC (**Figure 2**). After accounting for all covariates except serum albumin, we observed an inverted U-shaped correlation of serum albumin with BEOC. As well, we performed a threshold effect analysis to compare the single-line regression model with the two-segment regression model. The log-likelihood ratio was less than 0.05, indicating that model I (the one-line model) was statistically distinct from model II (the two-piecewise linear regression model). Given the statistical significance of the inflection point (K = 36), the two-piecewise linear regression model was deemed more appropriate, as indicated in **Table 3**. The highest point of BEOC was seen when the serum albumin reached 36 g/L.

**Table 3:** Threshold effect analysis of serum albumin with BEOC.

	β (95% CI) P value
Model I	
linear effect	-2.82 (-5.52, -0.13) 0.0486
Model II	
Inflection point (K)	36
Serum albumin< K	13.50 (1.71, 25.29) 0.0249
Serum albumin> K	-4.27 (-6.83, -1.71) 0.0011
Log likelihood ratio	0.0050

The model I and II controlled for all covariates.

### 3.4 Stratified connection of serum albumin with BEOC

Stratified analyses were carried out to evaluate the connection between serum albumin and BEOC in various subgroups. **Supplementary Table 2** displayed the results, stratified by sex, age, race, education, marriage, PIR, BMI, smoking, hypertension, diabetes, COPD, cancer, liver condition, and steroid use. We found that serum albumin was negatively linked to BEOC in women younger than 40, non-Hispanic Whites with low PIR, non-smokers, and people who did not have COPD, cancer, diabetes, liver disease, or use steroid. All stratified analyses showed no interaction (all p-values for interaction > 0.05).

### 3.5 XGBoost model

**Figure 3:**
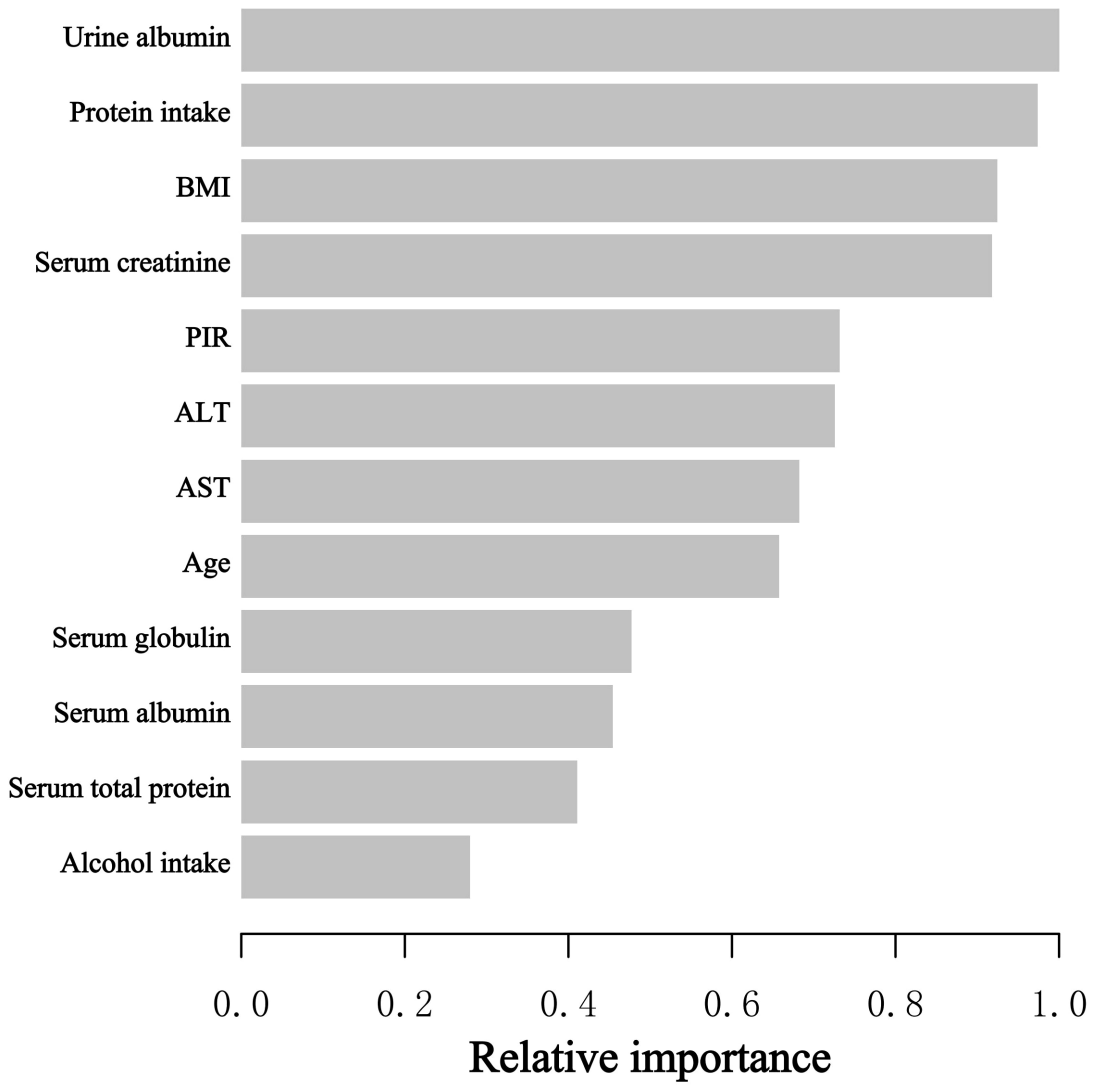
The XGBoost model provided the relative importance of each indicator on BEOC, along with the corresponding indicator importance score for every indicator.

When assessing the significance of the selected variable with regard to its impact on the BEOC, we adopted the XGBoost model. The selected variables included age, PIR, BMI, alcohol consumption, protein intake, AST, ALT, serum creatinine, serum albumin, serum globulin, serum total protein, and urine albumin. Ten variables, ranked in descending order of relative importance, primarily influenced the BEOC, according to the XGBoost model: urine albumin, protein consumption, BMI, serum creatinine, PIR, ALT, AST, age, serum globulin, and serum albumin (**Figure 3**).

## 4 Discussion

The investigation is the first and most extensive cross-sectional investigation to quantify the connection between protein intake, serum albumin, and BEOC in asthma patients, to the best of our knowledge. In our study, we found a positive correlation of protein intake with BEOC, but only in the univariable regression model. However, in univariate and multivariate regression models, there was a negative correlation of serum albumin with BEOC in asthma people. In addition, we utilized the GAM and threshold effect model to verify the link between serum albumin and BEOC, which exhibited an inverted U-shaped correlation. The maximal BEOC occurred at a serum albumin concentration of 36 g/L. The XGBoost model proved that urine albumin, protein consumption, BMI, serum creatinine, PIR, ALT, AST, age, serum globulin, and serum albumin are the top 10 influential factors affecting BEOC. Our investigation gave unique insights into the connection of serum albumin with BEOC in patients with asthma.

Serum albumin is the most abundant plasma protein in the body, managing the distribution of vascular fluid and maintaining plasma colloid osmotic pressure. And serum albumin also possesses powerful anti-inflammatory and antioxidant properties due to its multiple binding sites, which provide an ideal substrate for free radical removal (26). In addition, it can still bind diverse inflammatory mediators and regulate the immune response during systemic inflammation (27). It has been postulated that serum albumin is responsible for over 70 percent of the total free radical–trapping activity, making it the predominant antioxidant in the circulatory system. Besides, albumin also has various biologic functions, such as the binding and transport of endogenous and exogenous molecules, endothelial stabilization, anti-thrombotic effects, and so on (26). Consequently, serum albumin is also a valuable biomarker for a variety of illnesses, which include obesity, diabetes, rheumatoid arthritis, and carcinoma, while it can be employed to treat various diseases, such as shock, trauma, hemorrhage, acute respiratory distress syndrome, hemodialysis, acute liver failure, chronic liver disease, hypoalbuminemia, and so on (28).

Additionally, serum albumin is also a valuable biomarker for a variety of respiratory disorders, including lung cancer, chronic obstructive pulmonary disease, acute pulmonary embolism, and bronchiectasis (20, 29–31). Meanwhile, some studies have reported the role of albumin in asthma, too. A case-control study in Nigeria involving 37 asthma cases and 30 controls has discovered that children with asthma have significantly lower serum albumin concentrations in comparison to controls and that there is a negative association between serum IgE and serum albumin, but that there is no significant association between serum albumin and blood eosinophil counts or eosinophil percentage (32). According to a Turkish case-control study involving 40 asthma cases and 40 healthy subjects, there is a significant decrease in serum albumin concentration among bronchial asthma patients compared to controls (33). Likewise, a Japanese investigation has observed that the serum albumin levels of asthmatic children are substantially lower than those of children without asthma, whereas the levels of children with wheezing symptoms or a history of allergic diseases are not significantly different (34). And another cross-sectional study in Australia has found that serum albumin is lower in people with more severe asthma and is positively related to lung function (35). Similarly, Khatri SB et al. have discovered that plasma albumin levels are substantially lower in asthmatics compared to nonasthmatics and are positively correlated with lung function (% forced expiratory volume in 1 second) in asthmatics (36). And a prospective, multicenter study in Spain has demonstrated that the onset of asthma exacerbations is negatively correlated with serum albumin levels (37). However, AbdulWahab et al. have reported that there is no significant difference in serum albumin levels between asthmatic children and control groups (38). In the same way, another study in Turkey found that serum albumin levels are not significantly different between asthmatic children and control groups (39). As well, a few investigations have looked into the connection between protein consumption and asthma. Huang SL et al. reported that protein-rich and fat-rich foods of animal origin were associated with a higher prevalence of asthma in teenagers (40). However, Schwartz J. et al. found no connection between dietary protein consumption and wheezing or asthma in children in the USA (41). Another South Korean study proved that protein consumption was shown to be slightly but significantly connected with allergic rhinitis but not with asthma (42). And Han YY et al. reported no significant connection between protein food consumption and the prevalence of asthma in Puerto Rican kids (43). Due to the inclusion of various confounders in previous studies, inconsistent conclusions have been drawn. Our investigation found no independent connection between asthmatics’ protein consumption and BEOC. But serum albumin and BEOC had an inverted U-shaped association.

In contrast to prior research, ours has some advantages. First, our inquiry provides a relatively large, nationally representative sample of adult asthmatics. Secondly, because confounders may influence the results, we use stratified analysis to determine the relationship of serum albumin with BEOC in various populations. Then, we employ the XGBoost model to determine the relative significance of selected indicators on BEOC. Lastly, the inflection point in the nonlinear relationship of serum albumin with BEOC is determined by using GAM and a two-piecewise linear regression model. But we have to acknowledge the inquiry’s shortcomings. Cross-sectional studies cannot prove a causal link between serum albumin and BEOC. Additionally, there are certain potential confounding factors that we may disregard. The medications that influenced blood eosinophils in our investigation were predominantly cortisol medications and other anti-allergy medications, excluding biologics. Asthma diagnosis is dependent on questionnaires. Future research endeavors should explore the potential influence of serum albumin in regulating and managing asthma while also elucidating potential mechanisms of action.

## 5 Conclusion

Our investigation discovered there was no independent link between asthmatics’ protein intake and BEOC. However, we observed an inverted U-shaped relationship of serum albumin with BEOC, suggesting a possible correlation between the overall nutritional status of asthmatics and immune system changes. Our findings provide new directions for future research in the field of asthma management and therapy.

## Data availability statement

The datasets presented in this study can be found in online repositories. The names of the repository/repositories and accession number(s) can be found below: The official website of NHANES provides access to all available data (http://www.cdc.gov/nchs/nhanes/index.htm).

## Ethics statement

The studies involving humans were approved by National Center for Health Statistics’ Research Ethics Review Board. The studies were conducted in accordance with the local legislation and institutional requirements. Written informed consent for participation was not required from the participants or the participants’ legal guardians/next of kin in accordance with the national legislation and institutional requirements.

## Author contributions

JW: Conceptualization, Data curation, Formal analysis, Methodology, Writing – original draft. JX: Data curation, Formal analysis, Writing – original draft. QH: Data curation, Formal analysis, Writing – review & editing. MG: Data curation, Formal analysis, Writing – review & editing. SG: Conceptualization, Funding acquisition, Project administration, Supervision, Writing – review & editing.
