# Supplementary material for: Association between protein intake, serum albumin and blood eosinophil in US asthmatic adults
Source: Front Immunol. 2024 May 21;15:1383122. doi: 10.3389/fimmu.2024.1383122 (PMC11148351; doi:10.3389/fimmu.2024.1383122)
Supplement: Supplementary file 1 [file Table_1.docx]

Supplementary Material

| **Supplementary Table 1.** Association between protein intake and BEOC in asthmatics. | | | |
| --- | --- | --- | --- |
|  | Model A | Model B | Model C |
|  | β (95% CI) P value | β (95% CI) P value | β (95% CI) P value |
| Protein intake (gm) | 0.24 (0.05, 0.42) 0.0153 | 0.15 (-0.05, 0.35) 0.1359 | 0.17 (-0.03, 0.37) 0.1039 |
| Protein intake quartile |  |  |  |
| Q1 (1.26-51.02) | Reference | Reference | Reference |
| Q2 (51.06-71.76) | 8.56 (-11.17, 28.28) 0.3986 | 6.31 (-12.77, 25.40) 0.5196 | 9.42 (-8.44, 27.29) 0.3098 |
| Q3 (71.83-99.64) | 32.32 (4.50, 60.13) 0.0264 | 27.14 (-1.54, 55.82) 0.0693 | 25.50 (-1.94, 52.93) 0.0788 |
| Q4 (99.78-557.87) | 41.78 (16.68, 66.88) 0.0018 | 33.08 (6.32, 59.84) 0.0189 | 35.27 (10.35, 60.19) 0.0096 |
| P for trend | 0.0007 | 0.0129 | 0.0095 |
| Note: Model A controlled for none. Model B controlled for sex, age and race. Model C controlled for sex, age, race, education, marital status, PIR, BMI, smoking, alcohol intake, hypertension history, diabetes history, liver condition, COPD history, cancer history, steroid drugs use, AST, ALT, serum creatinine, serum albumin, serum globulin, serum total protein and urine albumin. Q1-Q4: Protein intake was grouped by quartile. | | | |

| **Supplementary Table 2.** Stratified associations between serum albumin and BEOC in asthmatics. | | | |
| --- | --- | --- | --- |
| Subgroup | N | β (95% CI) P value | P for interaction |
| Sex |  |  | 0.3341 |
| Male | 1031 | -1.20 (-5.99, 3.59) 0.6272 |  |
| Female | 1478 | -3.74 (-6.52, -0.95) 0.0133 |  |
| Age |  |  | 0.0588 |
| <40 | 985 | -3.92 (-7.35, -0.50) 0.0326 |  |
| 40-60 | 784 | -4.40 (-9.26, 0.45) 0.0861 |  |
| ≥60 | 740 | 1.62 (-2.79, 6.02) 0.4775 |  |
| Race |  |  | 0.9376 |
| Mexican American | 230 | -2.74 (-9.81, 4.34) 0.4553 |  |
| Other Hispanic | 264 | -2.49 (-12.57, 7.60) 0.6329 |  |
| Non-Hispanic White | 1089 | -3.52 (-6.59, -0.44) 0.0335 |  |
| Non-Hispanic Black | 617 | -1.25 (-6.92, 4.43) 0.6700 |  |
| Other Race | 309 | -0.07 (-7.10, 6.97) 0.9849 |  |
| Education |  |  | 0.8986 |
| Less than high school | 454 | -2.45 (-8.15, 3.25) 0.4057 |  |
| High school | 541 | -3.89 (-9.47, 1.69) 0.1825 |  |
| More than high school | 1514 | -2.56 (-5.62, 0.51) 0.1126 |  |
| Marital status |  |  | 0.7498 |
| Married | 1138 | -2.64 (-6.08, 0.81) 0.1441 |  |
| Single | 1161 | -2.66 (-6.79, 1.47) 0.2167 |  |
| Living with a partner | 210 | -4.89 (-10.37, 0.59) 0.0910 |  |
| PIR |  |  | 0.2818 |
| Low | 834 | -5.45 (-10.30, -0.59) 0.0359 |  |
| Middle | 838 | -0.12 (-4.52, 4.28) 0.9578 |  |
| High | 837 | -3.79 (-7.45, -0.13) 0.0519 |  |
| BMI |  |  | 0.7156 |
| <25 | 610 | -1.19 (-6.17, 3.79) 0.6443 |  |
| 25-30 | 704 | -3.42 (-8.20, 1.36) 0.1716 |  |
| ≥30 | 1195 | -3.53 (-7.24, 0.18) 0.0725 |  |
| Smoking |  |  | 0.2311 |
| Smoker | 1185 | -1.00 (-5.04, 3.03) 0.6292 |  |
| Non-smoker | 1324 | -4.27 (-7.82, -0.73) 0.0247 |  |
| Hypertension |  |  | 0.8114 |
| Yes | 1038 | -2.36 (-6.64, 1.91) 0.2873 |  |
| No | 1471 | -3.04 (-6.48, 0.40) 0.0939 |  |
| Diabetes |  |  |  |
| Yes | 415 | 2.57 (-6.36, 11.51) 0.5768 | 0.287 |
| No | 2014 | -3.63 (-6.49, -0.78) 0.0184 |  |
| Borderline | 80 | 3.33 (-9.26, 15.92) 0.6085 |  |
| COPD |  |  | 0.1556 |
| Yes | 263 | 3.81 (-6.38, 14.00) 0.4694 |  |
| No | 2246 | -3.57 (-6.24, -0.91) 0.0135 |  |
| Cancer |  |  | 0.5613 |
| Yes | 264 | -0.11 (-10.09, 9.87) 0.9830 |  |
| No | 2254 | -3.16 (-5.88, -0.43) 0.0307 |  |
| Liver condition |  |  | 0.3789 |
| Yes | 147 | 1.76 (-9.17, 12.68) 0.7549 |  |
| No | 2362 | -3.10 (-5.75, -0.45) 0.0291 |  |
| Steroid drugs use |  |  | 0.1097 |
| Yes | 416 | 2.44 (-4.97, 9.85) 0.5237 |  |
| No | 2093 | -3.72 (-6.43, -1.01) 0.0116 |  |
| Note: We adjusted the above analyses for sex, age, race, education, marriage, PIR, BMI, smoking, alcohol intake, protein intake, serum cotinine, history of hypertension, history of diabetes, liver condition, use of steroid drugs, AST, ALT, serum creatinine, serum globulin, serum total protein, and urine albumin. The above analyses were not adjusted for the stratification variable. | | | |
